# Supplementary material for: Quassinoid analogs with enhanced efficacy for treatment of hematologic malignancies target the PI3Kγ isoform
Source: Commun Biol. 2020 May 27;3:267. doi: 10.1038/s42003-020-0996-z (PMC7253423; doi:10.1038/s42003-020-0996-z)
Supplement: Supplementary file 1 — Reporting Summary [file 42003_2020_996_MOESM1_ESM.pdf]

nature research

Corresponding author(s): Erle S. Robertson

Last updated by author(s): Apr 23, 2020

Reporting Summary

Nature Research wishes to improve the reproducibility of the work that we publish. This form provides structure for consistency and transparency in reporting. For further information on Nature Research policies, see Authors & Referees and the Editorial Policy Checklist.

Statistics

For all statistical analyses, confirm that the following items are present in the figure legend, table legend, main text, or Methods section.

n/a	Confirmed	
☐	☒	The exact sample size (*n*) for each experimental group/condition, given as a discrete number and unit of measurement
☐	☒	A statement on whether measurements were taken from distinct samples or whether the same sample was measured repeatedly
☐	☒	The statistical test(s) used AND whether they are one- or two-sided<br>*Only common tests should be described solely by name; describe more complex techniques in the Methods section.*
☒	☐	A description of all covariates tested
☒	☐	A description of any assumptions or corrections, such as tests of normality and adjustment for multiple comparisons
☒	☐	A full description of the statistical parameters including central tendency (e.g. means) or other basic estimates (e.g. regression coefficient) AND variation (e.g. standard deviation) or associated estimates of uncertainty (e.g. confidence intervals)
☐	☒	For null hypothesis testing, the test statistic (e.g. *F*, *t*, *r*) with confidence intervals, effect sizes, degrees of freedom and *P* value noted<br>*Give P values as exact values whenever suitable.*
☒	☐	For Bayesian analysis, information on the choice of priors and Markov chain Monte Carlo settings
☒	☐	For hierarchical and complex designs, identification of the appropriate level for tests and full reporting of outcomes
☒	☐	Estimates of effect sizes (e.g. Cohen's *d*, Pearson's *r*), indicating how they were calculated

Our web collection on statistics for biologists contains articles on many of the points above.

Software and code

Policy information about availability of computer code

Data collection	The protocols have been clearly described in the "Methods" section, including Cytation 5 Multi-Mode Reader (BioTek), FACSCalibur System (Becton Dickinson), Orbitrap Fusion System (Thermo Fisher Scientific), StepOne Real-Time PCR System (Thermo Fisher Scientific), Odyssey Scanner (LiCor Inc.).
Data analysis	The analysis pipelines are introduced in the "Methods" section, which refer to FlowJo V10 (TreeStar), MaxQuant (Version 1.5.3.30), Odyssey Infrared Imaging System (Version 3.0.16), GraphPad Prism (Version 6.01).

For manuscripts utilizing custom algorithms or software that are central to the research but not yet described in published literature, software must be made available to editors/reviewers. We strongly encourage code deposition in a community repository (e.g. GitHub). See the Nature Research guidelines for submitting code & software for further information.

Data

Policy information about availability of data

All manuscripts must include a data availability statement. This statement should provide the following information, where applicable:

- Accession codes, unique identifiers, or web links for publicly available datasets
- A list of figures that have associated raw data
- A description of any restrictions on data availability

All data in this study have been included in this manuscript or submitted in to public datasets. Please refer to the "Data and materials availability" section.

Field-specific reporting

Please select the one below that is the best fit for your research. If you are not sure, read the appropriate sections before making your selection.

☒ Life sciences ☐ Behavioural & social sciences ☐ Ecological, evolutionary & environmental sciences

For a reference copy of the document with all sections, see nature.com/documents/nr-reporting-summary-flat.pdf

Life sciences study design

All studies must disclose on these points even when the disclosure is negative.

Sample size	Sample-size was calculated based on the similar study in the field and stated in the figure legends or the methods section.
Data exclusions	No data was excluded for analysis.
Replication	The replication numbers were indicated in the figure legends.
Randomization	Animals were randomly assigned for the different treatment in this study.
Blinding	The investigators were not blinded to group allocation during data collection and analysis.

Reporting for specific materials, systems and methods

We require information from authors about some types of materials, experimental systems and methods used in many studies. Here, indicate whether each material, system or method listed is relevant to your study. If you are not sure if a list item applies to your research, read the appropriate section before selecting a response.

Materials & experimental systems

n/a	Involved in the study	
☐	☒	Antibodies
☐	☒	Eukaryotic cell lines
☒	☐	Palaeontology
☐	☒	Animals and other organisms
☐	☒	Human research participants
☒	☐	Clinical data

Methods

n/a	Involved in the study	
☒	☐	ChIP-seq
☐	☒	Flow cytometry
☒	☐	MRI-based neuroimaging

Antibodies

Antibodies used	The detailed information of these antibodies has been introduced in this study and supplementary table 1.
Validation	All of the used antibodies are from the indicated commercial sources in the supplementary table 1.

Eukaryotic cell lines

Policy information about cell lines

Cell line source(s)	The source of all of the cell lines are described in the supplementary table 1.
Authentication	All of the cells were authenticated by the provider or our previous studies. No additional authentication was conducted in this study.
Mycoplasma contamination	The cell lines were tested to be mycoplasma-free.
Commonly misidentified lines (See ICLAC register)	No commonly misidentified lines were used.

Animals and other organisms

Policy information about studies involving animals; ARRIVE guidelines recommended for reporting animal research

Laboratory animals	NOD.CB17-Prkdcscid/J (NOD/SCID) mice

Wild animals | The study did not involve wild animals.

Field-collected samples | The study did not involve samples collected from the field.

Ethics oversight | These experiments were conducted under the supervision and management of the University of Pennsylvania Institutional Animal Care and Use Committee (IACUC) with approved protocol #804549.

Note that full information on the approval of the study protocol must also be provided in the manuscript.

Human research participants

Policy information about studies involving human research participants

Population characteristics | Human peripheral blood mononuclear cells (PBMCs), normal T-cells and B-cells were provided by the University of Pennsylvania Human Immunology Core (HIC), and Patient-derived xenografts (PDXs) were obtained from Fox Chase Cancer Center (FCCC).

Recruitment | These samples were from different unidentified and healthy donors with written, informed consent.

Ethics oversight | All the procedures were approved by the Institutional Review Board (IRB) at the University of Pennsylvania or the Fox Chase Cancer Center.

Note that full information on the approval of the study protocol must also be provided in the manuscript.

Flow Cytometry

Plots

Confirm that:

- [x] The axis labels state the marker and fluorochrome used (e.g. CD4-FITC).
- [x] The axis scales are clearly visible. Include numbers along axes only for bottom left plot of group (a 'group' is an analysis of identical markers).
- [x] All plots are contour plots with outliers or pseudocolor plots.
- [x] A numerical value for number of cells or percentage (with statistics) is provided.

Methodology

Sample preparation | The detailed protocol was described in the "Methods" section.

Instrument | FACSCalibur system (Becton Dickinson).

Software | FlowJo software V10 (TreeStar).

Cell population abundance | At least 10000 cells were collected per sample.

Gating strategy | Only living cells were used for gating.

- [ ] Tick this box to confirm that a figure exemplifying the gating strategy is provided in the Supplementary Information.
