# Supplementary material for: Quassinoid analogs with enhanced efficacy for treatment of hematologic malignancies target the PI3Kγ isoform
Source: Commun Biol. 2020 May 27;3:267. doi: 10.1038/s42003-020-0996-z (PMC7253423; doi:10.1038/s42003-020-0996-z)
Supplement: Supplementary file 2 — Supplementary Information [file 42003_2020_996_MOESM2_ESM.pdf]

Supplementary Method. Generation of the tested compounds in this study.

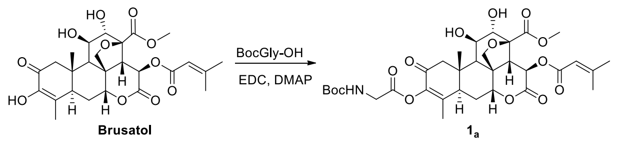

1_a (#15, 51-60-re-A). (1R,2S,3S,3aS,3a1R,4R,6aR,7aR,11aS)-methyl 9-(2-((tert-butoxycarbonyl)amino)acetoxy)-1,2-dihydroxy-8,11a-dimethyl-4-((3-methylbut-2-enoyl)oxy)-5,10-dioxo-2,3,3a,4,5,6a,7,7a,10,11,11a,11b-dodecahydro-1H-3,3a1-(epoxymethano) dibenzo[de,g]chromene-3-carboxylate

Boc-glycine (25 mg, 0.144 mmol), EDC·HCl (37 mg, 0.192 mmol), and 4-DMAP (12 mg, 0.0962 mmol) was added to brusatol (50 mg, 0.0962 mmol) in 2 mL THF. The reaction was stirred overnight. After diluting with EtOAc, the reaction mixture was washed with saturated aqueous NH_4Cl twice and then with brine once. The organic phase was concentrated and purified with HPLC with acetonitrile (0.1% TFA) in water (0.1% TFA) at a gradient of 34-69% in 10 min, to give a white solid (36.4 mg, 55.9%). ^{1}H NMR (300 MHz, $CDCl_3$): δ 5.59 (s, 1H), 4.78 (broad s, 1H), 4.69 (d, J = 7.5 Hz, 1H), 4.18-4.10 (m, 2H), 4.10-4.04 (m, 2H), 3.75-3.68 (m, 4H), 3.36-3.32 (m, 1H), 3.20-3.08 (m, 1H), 3.06-2.96 (m, 1H), 2.94-2.64 (m, 2H), 2.43-2.28 (m, 2H), 2.13 (s, 3H), 2.08-2.02 (m, 1H), 1.88 (s, 3H), 1.85-1.72 (m, 1H), 1.77 (s, 3H), 1.42 (s, 3H), 1.40 (s, 9H); Calculated for $C_{33}H_{43}NO_{14}$, 677.69; observed $(M+H)^+$ 678.8.

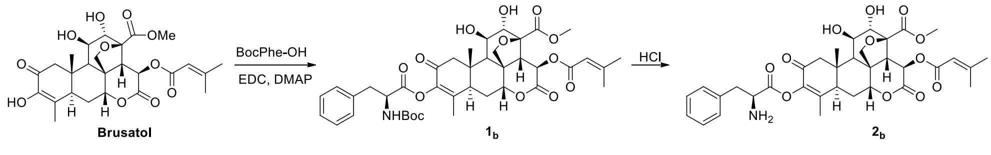

2_b (#14, 51-60-C). (1R,2S,3S,3aS,3a1R,4R,6aR,7aR,11aS)-methyl 9-(((S)-2-amino-3-phenylpropanoyl)oxy)-1,2-dihydroxy-8,11a-dimethyl-4-((3-methylbut-2-enoyl)oxy)-5,10-dioxo-2,3,3a,4,5,6a,7,7a,10,11,11a,11b-dodecahydro-1H-3,3a1-(epoxymethano)dibenzo[de,g]chromene-3-carboxylate.

According to the procedure for preparation of compound **1_a**, brusatol (20 mg, 0.0385 mmol) was treated with Boc-L-phenylalanine (15 mg, 0.0577 mmol), EDC·HCl (15 mg, 0.769 mmol), and 4-DMAP (4.7 mg, 0.0385 mmol) afford **1_b** [HPLC purification with a gradient of 41-76% of acetonitrile (0.1% TFA) in water (0.1% TFA) in 10 min], which was converted to **2_b** as a clear oil. upon acidic deprotection. ^{1}H NMR (300 MHz, $CDCl_3$): δ 7.34-7.26 (m, 5H), 5.576 (s, 1 H), 4.774 (s, 1 H), 4.656 (d, J = 7.5 Hz, 1 H), 4.48-4.32 (m, 1H), 4.44-4.34 (m, 1H), 4.16-4.06 (m, 2H), 3.70 (s, 3H), 3.62-3.52 (m, 2H), 3.32-3.26 (m, 2H), 3.22-2.95 (m, 1H), 2.95-2.84 (m, 2H), 2.43-2.25 (m, 2H), 2.11 (s, 3H), 2.07-2.00 (m, 1H), 1.86 (s, 3H), 1.75-1.63 (m, 3H), 1.62-1.56 (m, 1H), 1.42-1.16 (m, 3H); Calculated for $C_{35}H_{41}NO_{12}$, 667.70; observed $(M+H)^+$ 668.8.

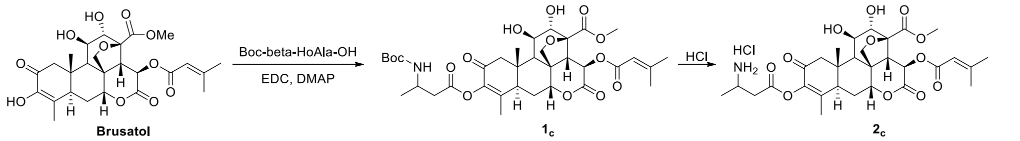

2_c (#26, 51-62-NB-B2, 51-69). (1R,2S,3S,3aS,3a1R,4R,6aR,7aR,11aS)-methyl 9-((3-aminobutanoyl)oxy)-1,2-dihydroxy-8,11a-dimethyl-4-((3-methylbut-2-enoyl)oxy)-5,10-dioxo-2,3,3a,4,5,6a,7,7a,10,11,11a,11b-dodecahydro-1H-3,3a1-(epoxymethano)dibenzo[de,g]chromene-3-carboxylate.

According to the procedure for preparation of compound **1_a**, brusatol (50 mg, 0.0962 mmol) was treated with Boc-β-HoAla-OH (29 mg, 0.144 mmol), EDC·HCl (37

mg, 0.192 mmol), and 4-DMAP (12 mg, 0.0962 mmol) afford **1c** [HPLC purification with a gradient of 34-69% of acetonitrile (0.1% TFA) in water (0.1% TFA) in 10 min], which was converted to **2c** upon acidic deprotection. There were two isomers in the mixture, which was purified by HPLC with a gradient of 20-55% in 10 min. The 2nd fraction was the main product, as a white solid (20.1 mg, 29.0% in two steps). ^{1}H NMR (300 MHz, $CDCl_3$): δ 5.59 (s, 1H), 4.79 (s, 1H), 4.68 (d, J = 8.1 Hz, 1H), 4.18-4.10 (m, 2H), 3.76-3.62 (m, 4H), 3.36-3.30 (m, 2H), 3.21-3.11 (m, 1H), 3.09-3.00 (m, 1H), 2.95-2.78 (m, 5H), 2.46-2.28 (m, 2H), 2.13 (s, 3H), 2.10-2.02 (m, 1H), 1.88 (s, 3H), 1.86-1.80 (m, 1H), 1.78 (s, 3H), 1.41 (s, 3H), 1.39 (s, 3H); Calculated for $C_{30}H_{39}NO_{12}$, 605.63; observed $(M+H)^+$ 606.8.

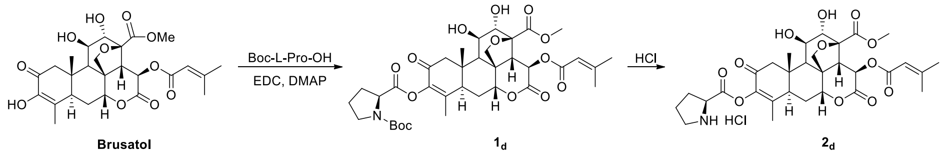

2d (#31, 51-64-NB, 51-80). (2S)-(1R,2S,3S,3aS,3a1R,4R,6aR,7aR,11aS)-1,2-dihydroxy-3-(methoxycarbonyl)-8,11a-dimethyl-4-((3-methylbut-2-enoyl)oxy)-5,10-dioxo-2,3,3a,4,5,6a,7,7a,10,11,11a,11b-dodecahydro-1H-3,3a1-(epoxymethano)dibenzo[de,g]chromen-9-yl pyrrolidine-2-carboxylate.

According to the procedure for preparation of compound **1a**, brusatol (20 mg, 0.0385 mmol) was treated with Boc-Pro-OH (12 mg, 0.0577 mmol), EDCI·HCl (15 mg, 0.769 mmol), and 4-DMAP (4.7 mg, 0.0385 mmol) afford **1d** [HPLC purification with a gradient of 35-70% of acetonitrile (0.1% TFA) in water (0.1% TFA) in 10 min], which was converted to **2d** as a clear oil (10.7 mg, 38.2%) upon acidic deprotection. ^{1}H NMR (300 MHz, $CDCl_3$): δ 5.59 (s, 1H), 4.79 (broad s, 1H), 4.71-4.64 (m, 2H), 4.17-4.09 (m, 2H), 3.75-3.65 (m, 5H), 3.64-3.54 (m, 2H), 3.36-3.30 (m, 2H), 3.20-3.70 (m, 2)2.58-2.44

(m, 2H), 2.44-2.28 (m, 2H), 2.13 (s, 3H), 2.18-2.02 (m, 3H), 2.00-1.92 (m, 2H), 1.88 (s, 3H), 1.84-1.74 (m, 2H), 1.64-1.40 (m, 3H); Calculated for $C_{31}H_{39}NO_{12}$, 617.64; observed $(M+H)^+$ 618.8.

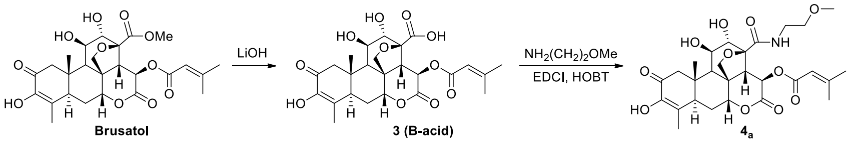

4_a (5, 51-57-C). (1R,2S,3S,3aS,3a1R,4R,6aR,7aR,11aS)-1,2,9-trihydroxy-3-((2-methoxyethyl)carbamoyl)-8,11a-dimethyl-5,10-dioxo-2,3,3a,4,5,6a,7,7a,10,11,11a,11b-dodecahydro-1H-3,3a1-(epoxymethano)dibenzo[de,g]chromen-4-yl 3-methylbut-2-enoate

3 (B-acid) (1R,2S,3S,3aS,3a1R,4R,6aR,7aR,11aS)-1,2,9-trihydroxy-8,11a-dimethyl-4-((3-methylbut-2-enoyl)oxy)-5,10-dioxo-2,3,3a,4,5,6a,7,7a,10,11,11a,11b-dodecahydro-1H-3,3a1-(epoxymethano) dibenzo[de,g] chromene-3-carboxylic acid (B-acid).

$LiOH_{(aq)}$ (1 M, 1.1 eq) was added to brusatol in THF, and the reaction was stirred overnight. The resulting intermediate was purified by HPLC with acetonitrile (0.1% TFA) in water (0.1% TFA). ^{1}H NMR ($CDCl_3$-MeOD): 6.33 (broad s, 1H, H15), 5.68 (s, 1H, H2'), 4.80 (s, 1H, H7), 4.74 (d, J = 7.6 Hz, 1H, H20b), 4.22 (s, 1H, H12), 4.13 (s, 1H, H11), 3.80 (d, J = 7.3 Hz, 1H, H14), 3.09 (d, J = 10.3 Hz, 1H, H20a), 2.98 (s, 1H, H1b), 2.93 (s, 1H, H5), 2.50-2.30 (m, 2H, H9 & H1b), 2.18 (s, 3H), 2.21-2.10 (m, 1H, H6a), 1.91 (s, 3H), 1.85 (s, 3H), 1.84-1.68 (m, 1H, H6b), 1.39 (S, 3H); Calculated for C25H30O11, 506.2; observed 507.6.

To the **3** (**B-acid**) (10 mg, 0.020 mmol) with excess triethylamine in 2 mL DCM, was added EDC·HCl (19 mg, 0.099 mmol), HOBT·H_2O (13 mg, 0.099 mmol), and 2-methoxyethanamine (9 μL, 0.099 mmol). The reaction was stirred at room temperature overnight. Then it was partitioned between EtOAc and saturated aqueous NH_4Cl. The organic phase was concentrated and purified by HPLC, resulting in the product as a white solid. ^1H NMR (300 MHz, $CDCl_3$): δ 5.59 (s, 1H), 4.78-4.68 (m, 2H), 4.14-4.09 (m, 1H), 3.91 (s, 1H), 3.71 (d, J = 7.5 Hz, 1H), 3.42-3.36 (m, 2H), 3.36-3.31 (m, 3H), 3.31-3.28 (m, 2H), 2.72-2.61 (m, 2H), 2.39-2.25 (m, 2H), 2.12 (s, 3H), 2.10-2.07 (m, 1H), 1.86 (s, 3H), 1.81-1.76 (m, 3H), 1.75-1.63 (m, 1H), 1.32 (s, 3H)); Calculated for $C_{28}H_{37}NO_{11}$, 563.59; observed $(M+H)^+$ 564.7.

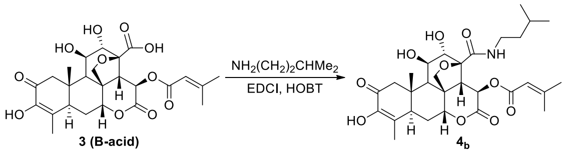

4_b (6, 51-57-D). (1R,2S,3S,3aS,3a1R,4R,6aR,7aR,11aS)-1,2,9-trihydroxy-3-(isopentylcarbamoyl)-8,11a-dimethyl-5,10-dioxo-2,3,3a,4,5,6a,7,7a,10,11,11a,11b-dodecahydro-1H-3,3a1-(epoxymethano)dibenzo[de,g]chromen-4-yl 3-methylbut-2-enoate.

According to the procedure for preparation of compound **4_a**, **3** (10 mg, 0.020 mmol) was treated with EDC·HCl (19 mg, 0.099 mmol), HOBT·H_2O (13 mg, 0.099 mmol), and isoamylamine (11 μL, 0.099 mmol) to afford **4_b** as a white solid. ^1H NMR (300 MHz, $CDCl_3$): δ 5.64 (s, 1H), 4.79 (d, J = 7.5 Hz, 1H), 4.73 (broad s, 1H), 4.19-4.15 (m, 1H), 3.93 (broad s, 1H), 3.71 (d, J = 7.5 Hz, 1H), 3.46-3.36 (m, 1H), 3.36-3.22 (m, 1H), 3.18-3.04 (m, 1H), 2.98-2.86 (m, 2H), 2.42-2.28 (m, 2H), 2.25-2.10 (m, 3H), 1.91-

1.86 (m, 4H), 1.75-1.65 (m, 3H), 1.64-1.50 (m, 2H), 1.41-1.36 (m, 2H), 1.35 (s, 3H), 0.92-0.85 (m, 7H)); Calculated for $C_{30}H_{41}NO_{10}$, 575.65; observed $(M+H)^+$ 576.8.

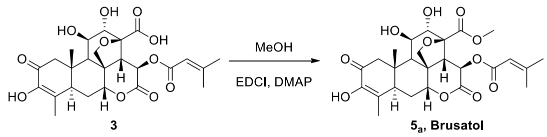

Brusatol (34, 51-70-A)

To **3** (15 mg, 0.030 mmol) in 2 mL MeOH, EDC·HCl (11 mg, 0.059 mmol) and 4-DMAP (4 mg, 0.030 mmol) was added. The reaction was stirred at room temperature overnight. Then it was partitioned between EtOAc and saturated aqueous NH_4Cl. The organic phase was concentrated down and purified by HPLC, resulting in the product as a white solid (4.9 mg, 32%). ^1H NMR (300 MHz, CD_3OD): δ 5.67 (s, 1H), 4.83 (broad s, 1H), 4.70 (d, J = 7.6 Hz, 1H), 4.22-4.14 (m, 2H), 3.74-3.68 (m, 4H), 3.40-3.20 (m, 2H), 3.01-2.92 (m, 1H), 2.88-2.79 (m, 1H), 2.57-2.48 (m, 1H), 2.35-2.25 (m, 1H), 2.23-2.19 (m, 1H), 2.17 (s, 3H), 1.94 (s, 3H), 1.89-1.85 (m, 1H), 1.84 (s, 3H), 1.37 (s, 3H); Calculated for $C_{25}H_{30}O_{11}$, 520.53; observed $(M+H)^+$ 521.7.

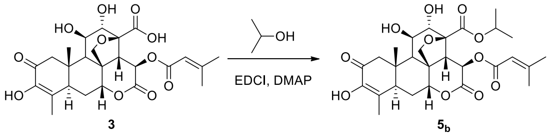

5_b (35, 51-70-C). (1R,2S,3S,3aS,3a1R,4R,6aR,7aR,11aS)-isopropyl 1,2,9-trihydroxy-8,11a-dimethyl-4-((3-methylbut-2-enoyl)oxy)-5,10-dioxo-2,3,3a,4,5,6a,7,7a,10,11,11a,11b-dodecahydro-1H-3,3a1-(epoxymethano)dibenzo[de,g]chromene-3-carboxylate.

According to the procedure for preparation of compound **5_a**, **3** (15 mg, 0.030 mmol) was treated with EDC·HCl (11 mg, 0.059 mmol) and 4-DMAP (4 mg, 0.030 mmol), and isopropanol (2 mL) to afford **5_b** as a white solid (3.5 mg, 21%). ^{1}H NMR (300 MHz, CD_3OD): δ 5.67 (s, 1H), 4.83-4.79 (m, 1H), 4.69 (d, J = 7.6 Hz, 1H), 4.21-4.14 (m, 2H), 3.75-3.66 (m, 1H), 3.40-2.20 (m, 3H), 3.02-2.92 (m, 1H), 2.88-2.80 (m, 1H), 2.58-2.48 (m, 1H), 2.35-2.25 (m, 1H), 2.25-2.19 (m, 1H), 2.16 (s, 3H), 2.00-1.89 (m, 4H), 1.89-1.80 (m, 3H), 1.37 (s, 3H), 1.33-1.22 (m, 6H); Calculated for $C_{28}H_{36}O_{11}$, 548.58; observed $(M+H)^+$ 549.7.

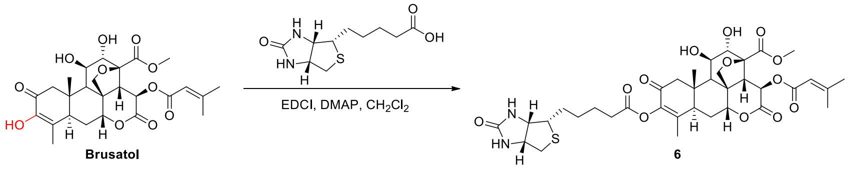

6 (51048). (1R,2S,3S,3aS,3a1R,4R,6aR,7aR,11aS)-methyl 1,2-dihydroxy-8,11a-dimethyl-4-((3-methylbut-2-enoyl)oxy)-5,10-dioxo-9-((5-((3aS,4S,6aR)-2-oxohexahydro-1H-thieno[3,4-d]imidazol-4-yl)pentanoyl)oxy)-2,3,3a,4,5,6a,7,7a,10,11,11a,11b-dodecahydro-1H-3,3a1-(epoxymethano)dibenzo[de,g]chromene-3-carboxylate.

To brusatol (20 mg, 0.0385 mmol) in 2 mL DCM, EDC·HCl (15 mg, 0.769 mmol), 4-DMAP (4.7 mg, 0.0385 mmol), and D-biotin (9 mg, 0.0385 mmol) was added. The reaction was stirred at room temperature for 2 days. Then it was diluted with EtOAc and washed with saturated aqueous NH_4Cl and brine. The organic phase was concentrated down and then purified by HPLC with acetonitrile (0.1% TFA) in water (0.1% TFA) at a gradient of 20-60% in 12 min, resulting in the product as a white solid (13.7 mg, 47.7%). ^{1}H NMR ($CDCl_3$-MeOD): (ppm) 5.62 (s, 1H, 2'), 4.78 (s, 1H, 15),

4.75-4.60 (m, 1H, H7), 4.55-4.35 (m, 2H, NHCH &H20b), 4.34-4.03 (m, 2H, NHCH & H12), 3.79 (s, 3H, OMe), 3.85-3.70 (m, 1H, H14), 3.30-3.00 (m, 3H, H20a & SCH & H11), 3.00-2.75 (m, 2H, SCHa & SCHb), 2.70-2.55 (m, 1H, H1a),2.45-2.00 (m, 5H, H5, COCH2 & CH3 & H1b), 1.98-1.30 (m, 18H, 3xCH2 & H6x2 & H9 & 3xCH3); Calculated for C36H46N2O13S, 746.3; observed 747.4.

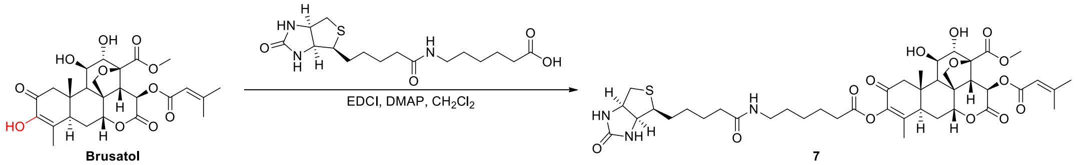

7 (51052). (1R,2S,3S,3aS,3a1R,4R,6aR,7aR,11aS)-methyl 1,2-dihydroxy-8,11a-dimethyl-4-((3-methylbut-2-enoyl)oxy)-5,10-dioxo-9-((6-(5-((3aS,4R,6aR)-2-oxohexahydro-1H-thieno[3,4-d]imidazol-4-yl)pentanamido)hexanoyl)oxy)-2,3,3a,4,5,6a,7,7a,10,11,11a,11b-dodecahydro-1H-3,3a1-(epoxymethano)dibenzo[de,g]chromene-3-carboxylate.

Brusatol (10.2 mg, 0.02 mmol), N-(+)-Biotinyl-6-aminohexanoic acid (7.0 mg, 0.02 mmol), EDCI (7.7 mg, 0.04 mmol), DMAP (2.4 mg, 0.04 mmol) were dissolved in CH_2Cl_2 (1 ml). The mixture was stirred under argon at room temperature for a week. Solvent was removed in vacuo, and the residue was partitioned between ethyl acetate and brine. The organic phase was concentrated. The desired conjugate (1.7 mg) was obtained as a white solid after HPLC separation (gradient: 20-53% of acetonitrile : water in 15 minutes) and lyophilization. ^{1}H NMR ($CDCl_3$-MeOD): (ppm) 5.69-5.63 (m, 1H, 2’), 4.86-4.81 (m, 1H, 7), 4.80-4.72 (m, 1H, H12), 4.58-4.48 (m, 1H, NH)4.34-4.27 (m, 2H, NH & H11), 4.27-4.22 (m, 1H, H20b), 3.78 (s, 3H, OMe), 3.82-3.72 (m, 1H, H14), 3.30-3.10 (m, 4H, H20a & NCH2 & SCH), 3.03-2.75 (m, 3H, H5 & 1a & SCHa), 2.65-2.35

(m, 2H, SCHb & 1b), 2.25-2.15 (m, 7H, 2xCOCH2 & CH3), 1.90 (s, 3H), 1.90-1.30 (m, 21H, 6xCH2 & H6x2 & H9 & 2xCH3); Calculated for C42H57N3O14S, 859.4; observed 861.0.

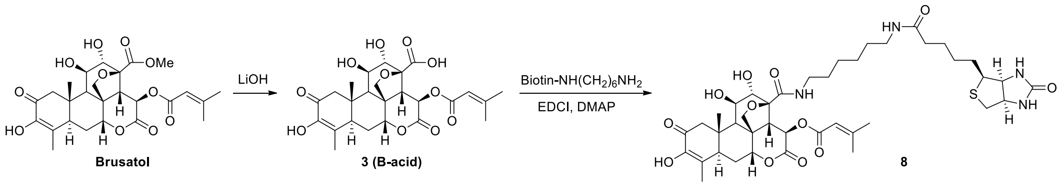

8 (51046). (1R,2S,3S,3aS,3a1R,4R,6aR,7aR,11aS)-1,2,9-trihydroxy-8,11a-dimethyl-5,10-dioxo-3-((6-(5-((3aS,4R,6aR)-2-oxohexahydro-1H-thieno[3,4-d]imidazol-4-yl)pentanamido)hexyl)carbamoyl)-2,3,3a,4,5,6a,7,7a,10,11,11a,11b-dodecahydro-1H-3,3a1-(epoxymethano)dibenzo[de,g]chromen-4-yl 3-methylbut-2-enoate.

To **3** (12 mg, 0.024 mmol) and excess triethylamine in 2 mL DCM, EDC·HCl (23 mg, 0.12 mmol), HOBT·H_2O (16 mg, 0.12 mmol), and N-(6-aminohexyl)-biotinamide (41 mg, 0.12 mmol) was added. The reaction was stirred at room temperature for 2 days. Then it was partitioned between EtOAc and saturated aqueous NH_4Cl. The organic phase was concentrated and then purified by HPLC at a gradient of 20-70% in 20 min to provide a white solid (3.7 mg, %) after lyophilization . ^{1}H NMR ($CDCl_3$-MeOD): 7.50-7.38 (m, 1H), 5.64 (s, 1H, H2’), 4.90-4.75 (m, 2H, H20b &H15), 4.55-4.45 (m, 1H, NHCH), 4.38-4.25 (m, 1H, NHCH), 4.23-4.15 (m, 1H, H7), 3.96 (s, 1H, H12), 3.85-3.75 (m, 1H, H11), 3.60-3.30 (m, 1H, H20a), 3.30-3.10 (m, 6H, 2xNHCH2 & SCH & H14), 3.00-2.85 (m, 3H, SCH2 & H5), 2.83-2.68 (m, 1H, H1a), 2.50-2.30 (m, 2H, H9 and H1b), 2.24-2.10 (m, 6H, CH3 & COCH2 & H6a), 1.93 (s, 3H), 1.85 (s, 3H), 1.85-1.20 (m, 18H, 7xCH2 & CH3 & H6b).

Supplementary Figures

a

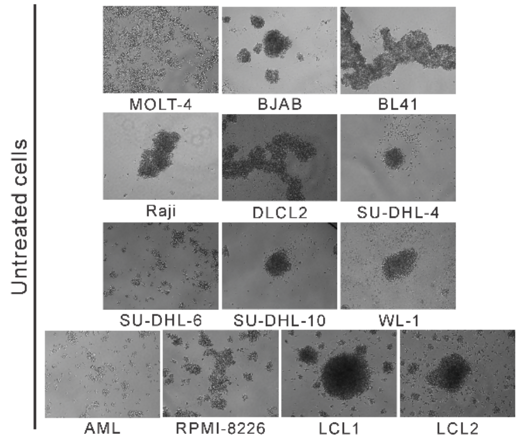

c

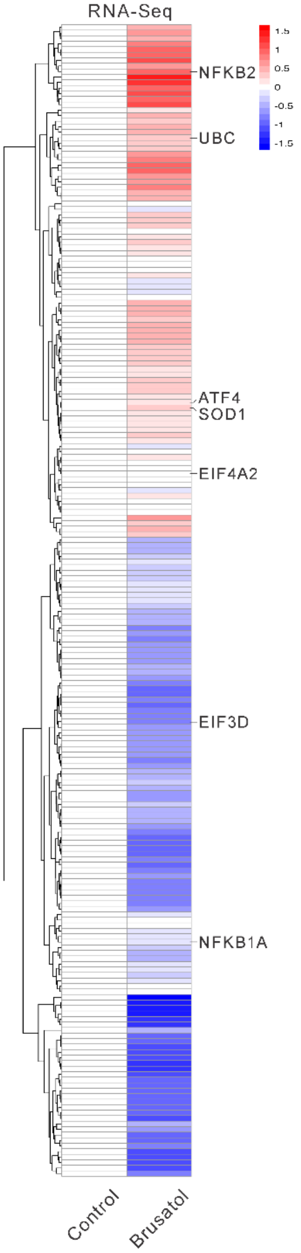

b

Name	p-value	Overlap
EIF2 Signaling	1.05E-35	65.2% (144/221)
Protein Ubiquitination Pathway	1.02E-18	50.6% (134/265)
mTOR Signaling	2.81E-17	53.2% (107/201)
Regulation of eIF4 and p70S6K Signaling	2.83E-16	56.1% (88/157)
B-Cell Receptor Signaling	6.40E-12	48.4% (92/190)

Supplementary Fig. 1. RNA-Seq analysis of Brusatol-treated cells. (a) Microscopic pictures show the normal status of tested cells. (b) P-value and overlap percent of Brusatol-related signaling pathways were demonstrated from RNA-Seq analysis. (c) Heatmap summarized the up-regulated and down-regulated genes in Brusatol-treated LCL1 cells.

a

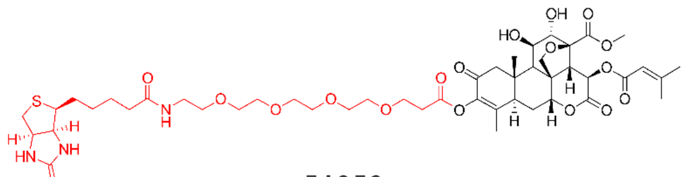

51053

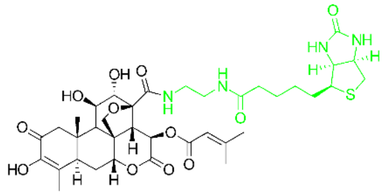

51045

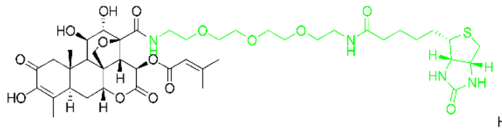

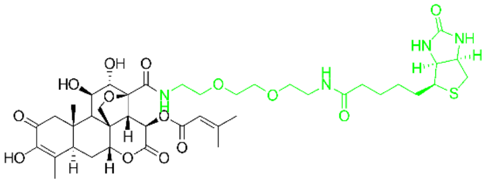

51047 **51050**

b

IC50(μM)	Modified at C-3 Position			Modified at C-21 Position			
	51048	51052	51053	51045	51046	51047	51050
PDX-129	1.726	0.076	0.656	>10	1.637	>10	>10
PDX-223	3.565	0.108	1.056	>10	8.974	>10	>10
PDX-255	0.789	0.025	0.249	>10	0.494	>10	>10
Toledo	>10	0.388	>10	>10	8.913	>10	>10
Pfeiffer	3.170	0.079	0.414	>10	3.013	>10	>10
JeKo-1	1.186	0.028	0.132	>10	1.393	>10	>10

Supplementary Fig. 2. Biotin-conjugated Brusatol compounds exhibit inhibitory effects. (a) Chemical structures of synthesized biotin-conjugated compounds were shown. (b) IC50 assays of biotin-conjugated compounds were performed in multiple PDXs and lymphoma cells.

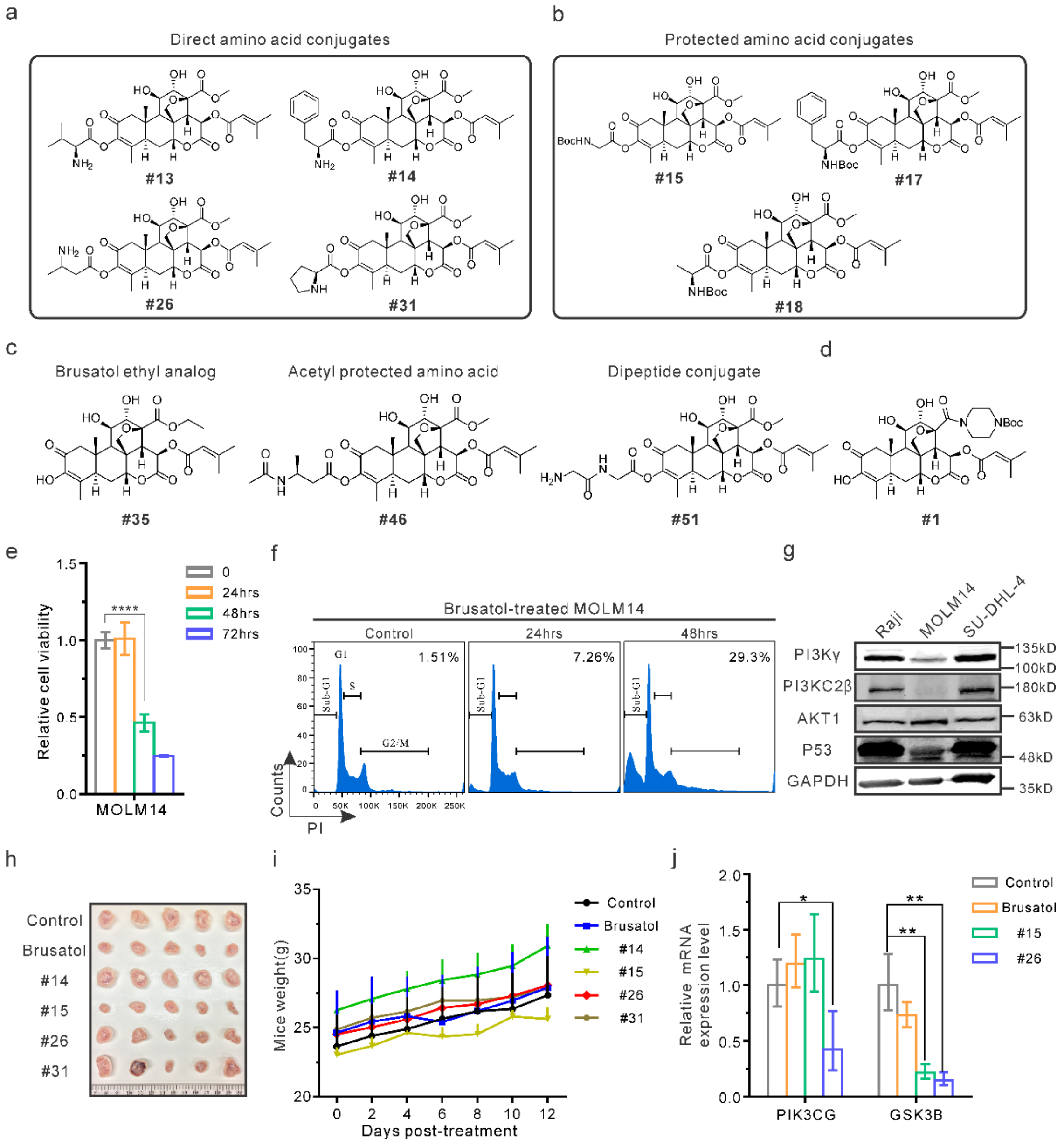

Supplementary Fig. 3. Developed Brusatol analogs show inhibitory effects *in vitro* and *in vivo*. (a-c) Chemical structures of representative Brusatol analogs with different modifications were shown. (d) The compound #1 was shown as an inactivated drug. (e) MOLM14 cells were treated with 100 nM Brusatol for different times (0, 24 hours, 48 hours, 72 hours) and cell viability was examined by detecting luminescence activity. Results are the mean ± standard error of the duplicates. ****$p < 0.0001$ shows the significant differences between the indicated groups. (f) MOLM14 cells were treated with 100 nM Brusatol for indicated times (0, 24 hours, 48 hours) and cell cycle assay was determined with flow cytometry. The subG1 population in cells was labeled as the percentage. (g) The expressions of PI3K associated proteins were detected in Raji, MOLM14, and SU-DHL-4 cells with western blot. (h) The microscopic examination of these tumors from the dissected mice was shown. (i) Mice weight was monitored after

Brusatol or its analogs treatment *in vivo*. (j) Total RNAs were extracted from the xenografts, and Real-time PCR analysis was performed to detect PIK3CG and GSK3B expression. Results are the mean ± standard error of the samples (n=3). $^{*}p < 0.05$ and $^{**}p < 0.01$ show the significant differences as compared to the control group.

Supplementary Table. Cell lines, antibodies, primers, and compounds used in this study.

**Cells**	Organism	Cell type/Disease	Source/Code
AML	human	Monocyte, acute myeloid leukemia	Dr. Mariusz Wasik
BJAB	human	B lymphocyte, Burkitt's lymphoma	Lab
BL41	human	Burkitt's lymphoma	Lab
DLCL2	human	Diffuse large B-cell lymphoma germinal center B-cell type, B-cell lymphoma	Dr. Ari Melnick
HepG2	human	Hepatocellular carcinoma	Lab
HL-60	human	Promyeloblast, acute promyelocytic leukemia	Dr. Jianxin You
H929	human	B lymphocyte, plasmacytoma, myeloma	ATCC (CRL-9068)
K562	human	Chronic myelogenous leukemia (CML)	Dr. Jianxin You
LCL1	human	Epstein-Barr virus-transformed lymphoblastoid cell lines	Lab
LCL2	human	Epstein-Barr virus-transformed lymphoblastoid cell lines	Lab
MOLM14	human	Acute myeloid leukemia	DSMZ (ACC 777)
MOLT-4	human	T lymphoblast, acute lymphoblastic leukemia	ATCC (CRL-1582)
NB-4	human	Acute promyelocytic leukemia	Dr. Jianxin You
PBMC	human	Primary Peripheral Blood Mononuclear Cells, normal	Upenn, HIC
Raji	human	B lymphocyte, Burkitt's lymphoma	Lab
RPMI-8226	human	Plasma cell myeloma, B lymphocyte, plasmacytoma	ATCC (CCL-155)
SU-DHL-4	human	Diffuse large B-cell lymphoma germinal center B-cell type, B lymphocyte	Dr. Ari Melnick
SU-DHL-6	human	Diffuse large B-cell lymphoma germinal center B-cell type, B lymphocyte	Dr. Ari Melnick
SU-DHL-10	human	Diffuse large B-cell lymphoma, B lymphocyte, large cell lymphoma	ATCC (CRL-2963)
WL-1	human	Mantle cell lymphoma	Dr. Mariusz Wasik

Toledo	human	B lymphocyte, diffuse large cell lymphoma; non-Hodgkin's B cell lymphoma	Dr. Wafik El-Deiry
Pfeiffer	human	Diffuse large cell lymphoma; non-Hodgkin's B cell lymphoma	Dr. Wafik El-Deiry
JeKo-1	human	Mantle cell lymphoma	Dr. Wafik El-Deiry
PDX-129	human	B-cell Follicular lymphoma	Dr. Wafik El-Deiry
PDX-223	human	Diffuse large B cell lymphoma	Dr. Wafik El-Deiry
PDX-255	human	Burkitt's lymphoma	Dr. Wafik El-Deiry
C17	human	EBV-containing nasopharyngeal carcinomas (NPC)	Dr. Paul Lieberman
NPC43	human	EBV-positive NPC cell line	Dr. Paul Lieberman
NPC53	human	EBV-positive NPC cell line	Dr. Paul Lieberman

**Antibodies**	Catalog No.	Company
anti-PI3Kα	sc-293172	Santa Cruz
anti-PI3Kβ	sc-376641	Santa Cruz
anti-PI3Kγ	sc-166365	Santa Cruz
anti-PI3Kδ	sc-55589	Santa Cruz
anti-PI3KC2β	sc-100407	Santa Cruz
anti-AKT1	sc-5298	Santa Cruz
anti-P53	sc-126	Santa Cruz
anti-GSK3	sc-7291	Santa Cruz
anti-CCND1	sc-8396	Santa Cruz
anti-GAPDH	sc-47724	Santa Cruz
anti-mTOR	#2983	Cell Signaling Technology

anti-Phospho-AKT(Ser473)	#9271	Cell Signaling Technology
**Primers /Targets**	Forward Sequences (5’-3’)	Reverse Sequences (5’-3’)
AKT1	TATTGTGAAGGAGGGTTG	ATTCTTGAGGAGGAAGTAG
ATF1	GGGACTTCAGACATTAACCATG	CAGGAGATGTCATCACCACA
ATF3	AAACAAGAAGAAGGAGAAGA	TTCAGTTCAGCATTCACA
ATF4	AGATAGGAAGCCAGACTA	CTCATACAGATGCCACTA
PIK3C2B	GAAGTATGAATGCTACCT	CAGTAACCAGAAGAAGTA
PIK3CG	GTGATTCTGGAAGCCTAT	CGATTACTTGGACTTGTTG
PIK3R4	TGCTATATTGCTCCTGAA	CTCCTCTTGTTCTCTGAT
FOXO1	CGAGTTATGGAGGTATGAG	GAGGAGAGTCAGAAGTCA
FOXO3	ACCATCCAAGAGAACAAG	TAAGTGAGTCCGAAGTGA
GSK3A	AAGGTTCTCCAGGACAAG	AAGTATCTCAGCCTCACAA
GSK3B	TATGAATTCCGCCATGTCAGGGCGGC	TATGCGGCCGCGGTGGAGTTGGAAG
NFκB	TTGCTGGTCCCACATAGTTG	ATGTATGTGAAGGCCCATCC
PTEN	ATCTAGGGGTAGAGGCAAGG	GTGGAGGACTGATGATGAAA
BAX	TGCTTCAGGGTTTCAAGGA	ACGGCGGCAATCATCCTCTG
TP53	GCTCGACGCTAGGATCTGAC	GCTTTCCACGACGGTGAC
TP73	CCCCATCAGGGGAGGTG	AGGGGACGCAGCGAAAC
JUP	AGTGTGCTGAAGATTCTG	TTGTTCTTGCTGTTGTTG
DPAGT1	CAATGCCATCAATATCCTA	AATCACCTTCCAACTCTA
NME2	GTTGGCAGGAACATCATT	ACCAGTTCTTCAGGCTTA

RNF8	GGTCTATTCCATTCATCAG	GTCTTCTTCAGTAACTTCAT
MYC	CATAGAATTCACCGCCATGCCC CTCA	CATAGCGGCCGCCGCACAAGA GTTC
GSTK1	CCGCAAAGGACTATACAT	AAGCATCACAGACAAGAA
HMOX1	GACTGCGTTCCTGCTCAA	CTCTGGTCCTTGGTGTCAT
NFE2L2	GTAGATGACAATGAGGTT	TGATTAGTAGCAATGAAGA
NQO1	GAGTCTGTTCTGGCTTAT	AACTGGAATATCACAAGGT
SOD1	TTAATCCTCTATCCAGAA	TCACAGAATCTTCAATAG
PIK3CG KO	CACCGGAACGGAGAAGAGATT CACG	AAACCGTGAATCTCTTCTCCGT TCC
PIK3CG KO verification	CGTGTTGCACCTTGTAAACTGG G	GAAGCTGAACTTTGCCCTTGGA C

**Compounds**	Catalog No.	Company
Copanlisib (BAY 80-6946)	S2802	Selleckchem
Duvelisib (IPI-145, INK1197)	S7028	Selleckchem
Idelalisib (CAL-101, GS-1101)	S2226	Selleckchem
IPI-549	S8330	Selleckchem
