# Supplementary material for: Quassinoid analogs with enhanced efficacy for treatment of hematologic malignancies target the PI3Kγ isoform
Source: Commun Biol. 2020 May 27;3:267. doi: 10.1038/s42003-020-0996-z (PMC7253423; doi:10.1038/s42003-020-0996-z)
Supplement: Supplementary file 6 — Description of Additional Supplementary Files [file 42003_2020_996_MOESM6_ESM.pdf]

Description of Additional Supplementary Files

File Name: **Supplementary Data 1**
Description: Upstream regulator analysis of RNA-Seq data in Brusatol-treated LCL1 cells by using Ingenuity Pathway Analysis (IPA) program (Excel file).

File Name: **Supplementary Data 2**
Description: Mass spectrometry data of three biotin-conjugated Brusatol analogs (Excel file).

File Name: **Supplementary Data 3**
Description: The source data in this manuscript (Excel file).
